# Involvement of ERK-Nrf-2 Signaling in Ionizing Radiation Induced Cell Death in Normal and Tumor Cells

**DOI:** 10.1371/journal.pone.0065929

**Published:** 2013-06-11

**Authors:** Raghavendra S. Patwardhan, Rahul Checker, Deepak Sharma, Santosh K. Sandur, Krishna B. Sainis

**Affiliations:** Radiation Biology & Health Sciences Division, Bio-Medical Group, Bhabha Atomic Research Centre, Trombay, Mumbai, India; Innsbruck Medical University, Austria

## Abstract

Prolonged oxidative stress favors tumorigenic environment and inflammation. Oxidative stress may trigger redox adaptation mechanism(s) in tumor cells but not normal cells. This may increase levels of intracellular antioxidants and establish a new redox homeostasis. Nrf-2, a master regulator of battery of antioxidant genes is constitutively activated in many tumor cells. Here we show that, murine T cell lymphoma EL-4 cells show constitutive and inducible radioresistance via activation of Nrf-2/ERK pathway. EL-4 cells contained lower levels of ROS than their normal counterpart murine splenic lymphocytes. In response to radiation, the thiol redox circuits, GSH and thioredoxin were modified in EL-4 cells. Pharmacological inhibitors of ERK and Nrf-2 significantly enhanced radiosensitivity and reduced clonogenic potential of EL-4 cells. Unirradiated lymphoma cells showed nuclear accumulation of Nrf-2, upregulation of its dependent genes and protein levels. Interestingly, MEK inhibitor abrogated its nuclear translocation suggesting role of ERK in basal and radiation induced Nrf-2 activation in tumor cells. Double knockdown of ERK and Nrf-2 resulted in higher sensitivity to radiation induced cell death as compared to individual knockdown cells. Importantly, NF-kB which is reported to be constitutively active in many tumors was not present at basal levels in EL-4 cells and its inhibition did not influence radiosensitivity of EL-4 cells. Thus our results reveal that, tumor cells which are subjected to heightened oxidative stress employ master regulator cellular redox homeostasis Nrf-2 for prevention of radiation induced cell death. Our study reveals the molecular basis of tumor radioresistance and highlights role of Nrf-2 and ERK.

## Introduction

Radiation therapy is an integral component of treatment of different types of solid cancers. Tumor cells possess inherent and/or exhibit acquired resistance to radiation induced cytotoxicity. Inherent radioresistance refers to constitutively active oncogenic, proliferative and/or anti-apoptotic signals, whereas acquired radioresistance refers to induction of pro-survival genes/proteins [1]. Exposure to clinically relevant doses of ionizing radiation induces multilayered signaling response in cancer cells by activating both cytoplasmic and nuclear signaling. Improved understanding of causes for constitutive and induced radioresistance in tumor cells may pave the way for designing effective treatment modality.

Ionizing radiation causes both direct and indirect damage to cells. Reactive oxygen species (ROS) generated as a result of indirect damage is the principal mediator of radiation induced damage to biological systems. Generation of ROS creates oxidative stress and disturbs redox balance within the cells [2]. Due to their high reactivity, electrophilicity and short lived nature they react with critical biomolecules in cell such as lipids, proteins and DNA [3]. This damage if unrepaired irreversibly commits cells to undergo apoptosis [4]. Cancer cells being metabolically active live in high oxidative stress environment [5], [6]. However, development of radioresistance in cancer cells would suggest that they have acquired the ability to eliminate the ROS and maintain a low steady state level. Effective elimination of ROS depends on how efficiently they are neutralized by antioxidants inside cells so that ionizing radiation induced damage is not permanently fixed. Our previous studies demonstrated that intrinsic radioresistance of lymphoma cells vis-à-vis normal lymphocytes may be due to lower basal and inducible ROS levels. Further, we have also shown that GSH levels and antioxidant enzyme activities were higher in lymphoma cells as compared to normal lymphocytes [4].

The levels of intracellular antioxidants and antioxidant enzymes are regulated by nuclear factor erythroid-2 related factor-2 (Nrf-2) [5]. It is a redox sensitive transcription factor, which belongs to a subset of basic leucine-zipper genes with a conserved cap “n” collar domain [7]. Under resting conditions, Nrf-2 is sequestered in cytoplasm by Keap1, an adaptor for Cul3-based E3 ubiquitin ligase that promotes constitutive proteasome mediated degradation of Nrf-2 [8]. Under oxidative stress, Nrf-2 is released from Keap1 and translocates to nucleus where it binds to antioxidant response element (ARE) in DNA and thereby induces transcription of a myriad antioxidant enzymes viz. catalase, Mn-superoxide dismutase, glutathione peroxidase, glutathione-S-transferase, hemeoxygenase I etc. [9]–[13].

Thus constitutive as well as inducible radioresistance of tumor cells may be due to one or more of the following: (1) hyperactive radical scavenging machinery [14], (2) induction of cell survival signaling molecules viz. Extracellular Related Kinase (ERK) and NF-kB, (3) induction of DNA damage repair [15]–[17] (4) cell cycle arrest and (5) increased anti-apoptotic proteins [15]. Many tumors have mutation in their EGF receptor leading to sustained proliferation stimulus via activation of ERK [18]–[20] that controls cell proliferation and survival directly by activating transcription factors like ets1 and Nrf-2. It is important to identify potential targets to sensitize tumors to radiotherapy without significant collateral damage to normal tissues. Thus it is required to identify molecular targets that are differentially expressed between normal and tumor cells. Numerous reports have highlighted the importance of targeting pro-inflammatory pathways and its regulatory transcription factor NF-kB to enhance radiosensitivity of tumor cells [21]–[25]. Very little attention is paid to other molecular targets important in tumor radioresistance.

Nrf-2 activation is implicated in anti-inflammatory activity [26]–[30] and chemopreventive studies [31]–[33]. Recent reports indicate that ionizing radiation activates Nrf-2 pathway and targeting this pathway may impact outcome of radiation therapy [34], [35]. Further, we recently showed that upregulation of Nrf-2 in lymphocytes augmented their radioresistance in vitro and also prevented radiation induced morbidity and mortality in mice [36]. Based on these reports, we hypothesized that activated ERK and Nrf-2-ARE pathway [37] may contribute to the constitutive and inducible radioresistance in tumor cells vis-à-vis normal cells. To probe the molecular mechanism of differential radiosensitivity between tumor and normal cells, we compared murine splenic lymphocytes from C57/BL6 mice and syngenic murine T cell lymphoma cells (EL-4). The aim of this study is to determine the contribution of ERK/Nrf-2-ARE pathway in tumor radioresistance.

## Materials and Methods

### Chemicals

RPMI-1640, HEPES, EDTA, EGTA, PMSF, leupeptin, aprotinin, benzamidine, dithiothreitol (DTT), NP40, propidium Iodide (PI), dimethyl sulfoxide (DMSO) dihydrodichlorofluoresceindiacetate (DCFDA), dihydroethidium (DHE) and dihydrorhodamine 123 (DHR123), Trizol reagent, DNase I amplification grade, cDNA synthesis kit and N-Amp SYBR green PCR mix were purchased from Sigma Chemical Co. (MO, USA). Fetal calf serum (FCS) was obtained from GIBCO BRL (MD, USA). Pharmacological inhibitors of Nrf-2, MEK, P38, JNK, thioredoxin reductase, hemeoxygenase I were purchased from Calbiochem (CA, USA). Inhibitory peptide for NF-κB was purchased from IMGENEX (San Diego, CA). Plasmid miniprep kit and lipofectamine 2000 were purchased from Invitrogen (Grand Island, NY). Carboxymethylcellulose based medium was purchased from R&D system (Minneapolis, USA). Monoclonal antibody against CD3, CD28 and HO-1(PE labeled) was procured from BD Pharmingen (CA, USA). Antibodies against IκBα, HO-1, Mn-SOD, ERK, p-ERK and α-tubulin were obtained from Cell Signaling Technologies (CA, USA). shRNA containing plasmids for ERK and Nrf-2 were purchased from Origene, Rockville, MD, USA. Thioredoxin activity kit was purchased from IMCO, Sweden. Polynucleotide kinase, kinase buffer for EMSA were purchased from New England Biolabs. Oligonucleotide probes for NF-kB and Nrf-2 were purchased from Santacruz Biotechnology (CA, USA). All other chemicals used in our studies were obtained from reputed manufacturers and were of analytical grade.

### Animal Maintenance

Six to eight week old inbred C57/BL6 male mice, weighing approximately 20–25 g, reared in the animal house of Bhabha Atomic Research Centre were used. They were housed at constant temperature (23°C) with a 12/12 hour light/dark cycle and were given mouse chow and water ad libitum. The Institutional Animal Ethics Committee of Bhabha Atomic Research Centre, Government of India, has approved the animal studies and the guidelines issued by the ethics committee regarding the maintenance and dissections of small animals were strictly followed. Project No. BAEC/08/11 and Date of approval: October, 2011.

### Cell Culture

EL-4 cell line (Murine T cell lymphoma) was obtained from Health Protection Agency Culture Collections, UK Cat. No. 85023105 and cultured in RPMI 1640 supplemented with 10% fetal calf serum, 100 U/ml penicillin and 100 µg/ml streptomycin. Cells were incubated at 37°C in a 5% CO_2_ humidified atmosphere in a CO_2_ incubator. Splenic lymphocytes were isolated from C57BL/6 mice by gently squeezing the spleen against 100 µ nylon mesh. The RBCs were lysed by hypotonic shock and viable cells were counted by trypan blue dye exclusion. One million cells were stimulated with 1 µg/ml plate bound mouse CD3 antibody and 1 µg/ml soluble mouse CD28 antibody in 24 well plates. Then cells were cultured in RPMI 1640 medium supplemented with 10% fetal calf serum, 100 U/ml penicillin and 100 µg/ml streptomycin for 72 h.

### Exposure to Ionizing Radiation

^60^Co was used as source of γ-irradiation and cells were given a dose of 4Gy in medium without FCS at a dose rate of 2.5 Gy/min.

### Estimation of Apoptosis (Propidium Iodide Staining and DNA Ladder Assay)

The percentage of apoptotic cells was estimated using a flowcytometer (Cyflow, Partec) [38]. DNA ladder assay was performed to confirm apoptosis. EL-4 cells (5×10^5^) or murine splenic lymphocytes were exposed to 4 Gy ionizing radiation and cultured for 24 h/48 h in RPMI 1640 medium supplemented with 10% fetal calf serum (FCS) in a 5% CO_2_ atmosphere. Unirradiated cells served as control. The cells were washed with PBS and incubated with 1 ml of staining solution containing 50 µg/ml propidium iodide, 0.1% sodium citrate and 0.1% triton X-100 overnight [38]. A total of 20,000 cells were acquired in a flowcytometer and analyzed using FloMax® software. The pre G_1_ population represented the apoptotic cells. The cells were washed with PBS and processed for DNA fragmentation as described earlier [39]. Briefly, cells were lysed in mammalian cell lysis buffer (10 mM Tric-Cl, 100 mM EDTA, 0.5% SDS & 100 µg/ml RNase A). The lysate was centrifuged at 12000 g/4°C/20 min, supernatants were transferred to new tube and incubated with RNase A followed by proteinase K. Phenol/chloroform/isoamyl alcohol extraction was performed. The aqueous phase was collected after centrifugation in a new tube. Two volumes of 100% chilled ethanol and 1/10^th^ volume of 3 M sodium acetate (pH 5.2) were added and DNA was allowed to precipitate. After centrifugation the pellet was washed with 70% ethanol, air dried and dissolved in deionized DNase free water. Samples were run on 1.2% agarose gel at 60 V for 2 h and DNA ladder was visualized using Gel Documentation System (DNR Biosystem).

### Intracellular ROS Measurements

To detect intracellular ROS, EL-4 and murine splenic lymphocytes were incubated with 20 µM oxidation-sensitive dichlorofluoresceindiacetate (DCF-DA) [40] or 5 µM dihydroethidium or 5 µM dihydrorhodamine 123 for 25 min at 37°C [4] before exposure to 4 Gy ionizing radiation. Increase in fluorescence resulting from oxidation of H_2_DCF to DCF (485 nm excitation/535 nm emission) or DHE to hydroethidium (480 nm excitation/610 nm emission) or DHR to rhodamine was (500 nm excitation/536 nm emission) measured using a spectrofluorimeter.

### Intracellular GSH Assay

GSH/GSSG ratio was measured by conventional enzyme cycling method [41].

### Thioredoxin Activity

EL-4 cells were exposed to dose of 4 Gy radiation and thioredoxin activity was measured in them using Thioredoxin Activity Kit (IMCO, Sweden) as per manufacturer’s instructions.

### shRNA Knockdown of EL-4 Cells

Two separate shRNA plasmids were tested for knocking down for either Erk or Nrf-2. The most effective plasmids (Cat. No. TF515053 for Nrf-2 and TF502598 for Erk Origene) were used in subsequent experiments. The cells (4×10^5^) were seeded in 800 ul medium free of antibiotic and FCS in a 6 well plate. For each transfection, DNA (1 µg): lipofectamine 2000 (10 µg) complex was prepared separately and incubated for 45–60 min at RT and added to. Cells were further cultured for 48 hrs for transgene expression.

### Clonogenic Assay

EL-4 cells were seeded at cell density of 1000 cells per well in mouse complete methylcellulose medium with or without exposure to ionizing radiation 4 Gy. Cultured for 15 days and observed for colony formation. Cells were pretreated with pharmacological inhibitors of ERK (10 µM U0126) or JNK (10 µM JNK II) or p38 (10 µM SB 202190) or Nrf-2 (5 µM ATRA) or HO-1 (5 µM SNPP) or TrxRd (25 nM Auranofin) or Ras (10 µM Farnesyl Thiosalicylic Acid) for 2 h prior to exposure to ionizing radiation. Cells were spun down, supernatant was discarded and resuspended in carboxymethylcellulose medium. Cells were cultured in six well plates with one well containing water to keep the medium moist and prevent from drying.

### RNA Isolation, cDNA Synthesis and Quantitative Real Time PCR

mRNA levels in the samples were quantified by quantitative real-time polymerase chain reaction (qPCR) as described previously [42], [43]. Breifly, 10×10^6^ (murine splenic lymphocytes) or 2×10^6^ EL4 cells were exposed to ionizing radiation 4 Gy and harvested at different time intervals (2, 6, 12, 24 h). Cells were processed for RNA isolation by homogenizing in trizol reagent and vortexed after adding chloroform. Cells were incubated for 5 min at RT and then centrifuged at 14000 rpm for 15 min at 4°C. Supernatant was collected in new tube and was allowed to precipitate by adding equal volume of isopropanol at RT for 20 min. Cells were spun down at 14000 rpm for 15 min at 4°C, supernatant was discarded and pellet was washed by chilled 70% ethanol, air dried and dissolved in RNase free water. Purity and quantity of RNA was estimated in a 96-well quartz plate using Synergy H1 Hybrid reader (Biotek) at 260 and 280 nm. 1 µg RNA was used to prepare cDNA using cDNA synthesis kit (Sigma) as per manufacturer’s instructions. Different dilutions of cDNA were made to calculate the efficiency of real time PCR. Real time PCR for Nrf-2, HO-1, GCLC, SOD2, catalase, thioredoxin reductase I was performed using respective forward and reverse primers (Table 1) and 2× SYBR mix from Sigma. Melting Temperature was set at 95°C, cycling conditions were 95°C-20 sec, 58°C-20 sec, 72°C-30 sec (40 cycles) and extension was carried out at 72°C-5 min. The expressions of genes were normalized against that of a housekeeping gene, β-actin, and plotted as relative change in the expression with respect to control. Sequence of forward and reverse primers used for RT-PCR is provided in table 1.

**Table 1:** 

Gene	Sequence
**HMOX-1**	Forward: AGGTACACATCCAAGCCGAGA Reverse: CCATCACCAGCTTAAAGCCTT
**TrxRd1**	Forward: GGGTCCTATGACTTCGACCTG Reverse: AGTCGGTGTGACAAAATCCAAG
**GCLC**	Forward: CTACCACGCAGTCAAGGACC Reverse: CCTCCATTCAGTAACAACTGGAC
**Nrf-2**	Forward: CTTTAGTCAGCGACAGAAGGAC Reverse: AGGCATCTTGTTTGGGAATGTG
**Catalase**	Forward: AGCGACCAGATGAAGCAGTG Reverse: AGGACATCAGGTCTCTGCGA
**Mn-SOD**	Forward: CAGACCTGCCTTACGACTATGG Reverse: CTCGGTGGCGTTGAGATTGTT
**β-actin**	Forward: GCGGGAAATCGTGCGTGACATT Reverse: GATGGAGTTGAAGGTAGTTTCGTG

### Analysis of Proteins by Flow Cytometry

One million EL-4 cells were exposed to a dose of 4 Gy radiation and harvested at 6, 12 and 24 hrs post irradiation. Cultured cells were fixed with 4% paraformaldehyde for 20 min at 4°C. Before staining with monoclonal antibody against HO-1, cells were permeabilized with PBST (PBS containing 0.02% Tween-20) thrice for 5 min each at room temp. The cells were washed two times with wash buffer, blocked with blocking buffer and then incubated with the indicated mAbs for 30 min at room temp, washed twice and analyzed using a Partec Cyflow flowcytometer [40].

### Western Blot Analysis

EL-4 cells (2×10^6^/group) were exposed to 4 Gy radiation and harvested at different time intervals. Cytosolic or nuclear extract was prepared as described earlier [44]. Equal amounts of protein (50 µg) from cytosolic extracts were resolved by SDS-PAGE (10%), transferred to nitro cellulose membrane, blocked with 5% milk and probed with the primary antibodies specific to IκB-α or Mn-SOD or HO-1. Further, blots were washed with 1× TBST (50 mM Tris-HCl, pH7.4, 150 mM NaCl, 0.1% Tween 20) and incubated with horseradish peroxidase-labeled secondary antibody for 1 h. The membranes were washed, and specific bands were visualized on X-ray films using enhanced chemiluminiscence kit (Roche, Germany).

### Electrophoretic Mobility-shift Assay (EMSA)

EL-4 cells were exposed to 4 Gy and cells were harvested at different time intervals and nuclear pellets were prepared as described earlier [45]. EMSA was performed by incubating 10 µg of nuclear proteins with 16 fmol of ^32^P-end-labeled, 45-mer double-stranded NF-κB oligonucleotides from the human immunodeficiency virus long terminal repeat (5′-TTGTTACAAGGGACTTTCCGCT***GGGGACTTTC*** *C*AGGGAGGCGTGG-3′; italic indicates NF-κB binding sites) and Nrf-2 oligonucleotide (5′-TGGGGAACCTGTGCTGATCACTGGAG-3′) in the presence of 0.5 µg of poly(2′-deoxyinosinic–2′-deoxycytidylic acid) in binding buffer (25 mM Hepes, pH 7.9, 0.5 mM EDTA, 0.5 mM DTT, 1% NP-40, 5% glycerol, and 50 mM NaCl) for 30 min at 37°C. The DNA–protein complex formed was separated from free oligonucleotide on 6.6% native polyacrylamide gels using buffer containing 50 mM Tris, 200 mM glycine, and 1 mM EDTA, pH 8.5.The dried gel was exposed to a phosphorimage plate and the radioactive bands were visualized using a phosphorimage plate scanner (Fuji).

### Statistical Analysis

Data are presented as mean ± SEM from three replicates in each experiment. For each parameter three independent experiments were carried out. The statistical analysis was done using Student’s t-test using Microcal Origin 6.0 software. *refers to p<0.05, as compared to control and # refers to p<0.05, as compared to irradiated cells.

## Results

### EL-4 Cells were more Resistant to Ionizing Radiation Induced Cell Death as Compared to Normal as well as Activated Murine Splenic Lymphocytes

**Figure 1:**
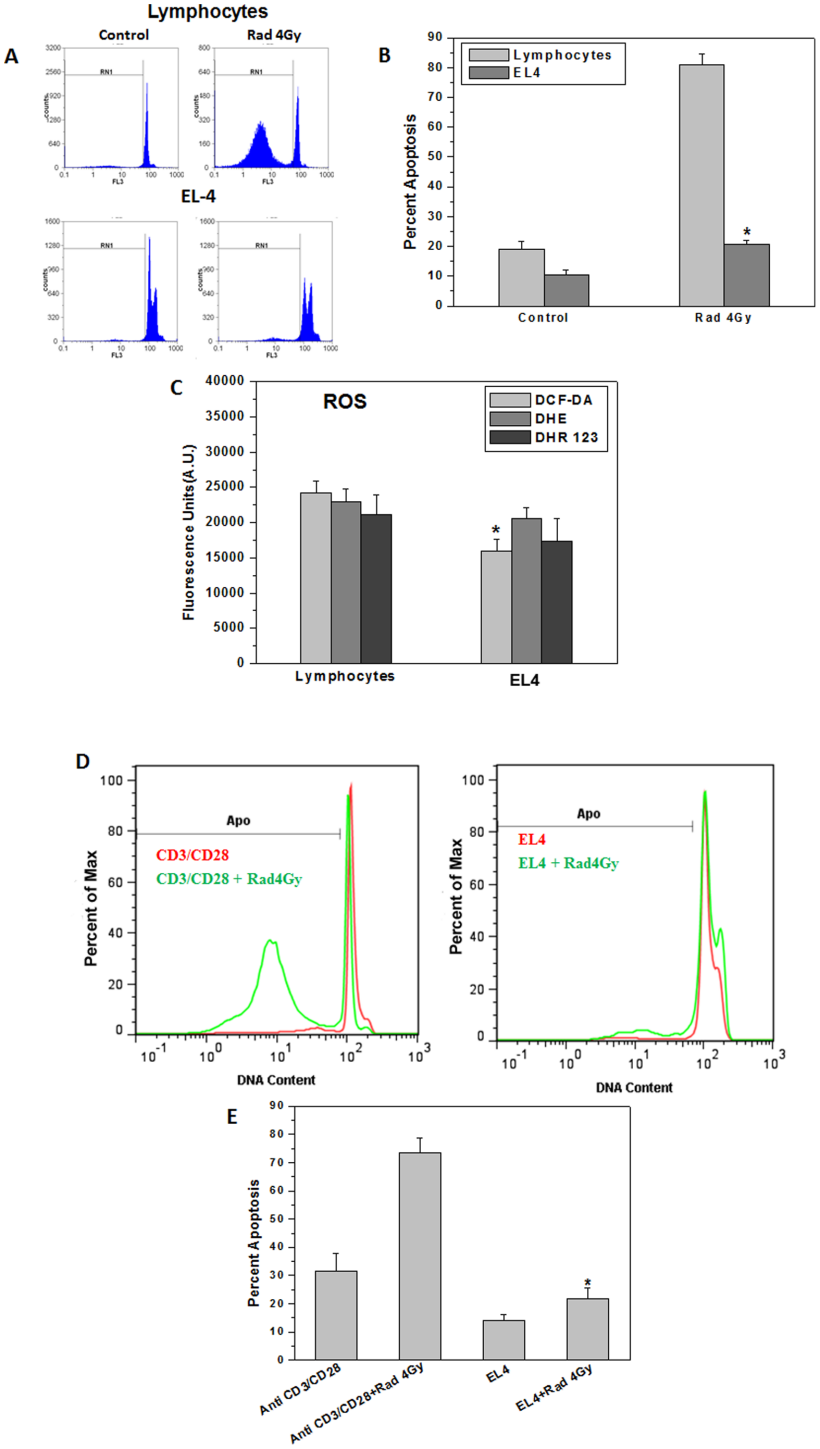
EL-4 cells were more resistant to ionizing radiation induced cell death as compared to normal murine lymphocytes. (A) Flow cytometric profile of propidium iodide stained normal murine splenic lymphocytes cultured for 24 h post 4 Gy irradiation. Pre-G1 peak (gate RN1) represents apoptotic population. (B) Bar graph represents percent apoptotic cells. Data points represent mean±S.E.M. from nine replicates from three independent experiments. *p<0.01, as compared to irradiated lymphocytes. (C) ROS levels in normal splenic lymphocytes and EL-4 cells were estimated by staining with DCF-DA (20 µM), DHE or DHR123 (5 µM each) 30 min at 37°C followed by fluorescence emission measured at their respective wavelength. Bar graph shows relative fluorescence units indicating ROS levels, mean±S.E.M. from nine replicates from three independent experiments. *p<0.01, as compared to normal lymphocytes. (D&E) One million splenic lymphocytes stimulated with anti-CD3/anti-CD28 antibodies for 72 h in 24-well plate or EL-4 cells were exposed to ionizing radiation (4 Gy) and cultured for 24 h. Cells were stained with propidium iodide and acquired on flowcytometer. Data points represent mean±S.E.M. from nine replicates from three independent experiments. *p<0.01, as compared to activated and irradiated lymphocytes.

Fig. 1 shows IR induced apoptosis in mouse T lymphoma cell line EL-4 vs. resting and activated mouse splenic lymphocytes. The cells were exposed to 4 Gy and cultured for 24 h in medium containing 10% FCS in a CO_2_ incubator at 37°C and apoptosis was measured by propidium iodide staining followed by flowcytometric analysis (Fig. 1A&B). Ionizing radiation induced apoptosis in about 60% of murine splenic lymphocytes. EL-4 lymphoma cells showed significantly lower radiation induced apoptosis (∼10%) as compared to murine lymphocytes. Basal levels of cellular ROS (hydroxyl, superoxide and H_2_O_2_) were significantly lower in tumor cells as compared to their normal counterpart (Fig. 1C). The basal levels of intracellular ROS in CD3/CD28 stimulated lymphocytes were significantly higher than those in EL-4 cells (data not shown). Normal lymphocytes are non-proliferating. Hence antibody stimulated lymphocytes were used as additional control. Anti CD3/CD28 stimulated lymphocytes exhibited significantly higher cell death as compared to EL-4 cells in response to radiation (Fig. 1D&E).

### Murine T cell Lymphoma Cells have Active Redox Circuits

**Figure 2:**
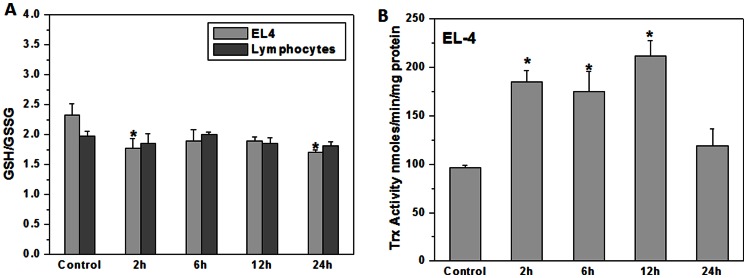
Murine T cell lymphoma showed active redox circuits as compared to normal lymphocytes. (A) Intracellular GSH levels were measured by conventional enzyme cycling method [41] at 2, 6, 12 or 24 h post irradiation (4 Gy) in normal lymphocytes and EL-4 lymphoma cells. Bar graph represents GSH/GSSG ratio in normal murine splenic lymphocytes and EL-4 cells. Data points represent mean±S.E.M. from nine replicates from three independent experiments. *p<0.05 as compared to untreated lymphoma cells (B) Thioredoxin activity in wild type EL-4 cells at 2, 6, 12 & 24 h post irradiation (4 Gy) was measured. Bar diagram shows thioredoxin activity in EL-4 cells. Each bar shows mean±S.E.M from nine replicates from three independent experiments. *p<0.01 as compared to untreated lymphoma cells.

Basal and ionizing radiation induced levels of GSH were estimated in normal lymphocytes and lymphoma cells by conventional enzyme cycling method. There was a significant decrease in GSH/GSSG ratio post-irradiation in normal as well as tumor cells at all the time points (Fig. 2A). EL-4 cells showed increase in thioredoxin activity from 2 to 12 h after exposure to 4 Gy radiation which correlated with their higher radioresistance (Fig. 2B).

### ERK or Nrf-2 Inhibitor Significantly Enhanced Radiosensitivity of Tumor Cells

**Figure 3:**
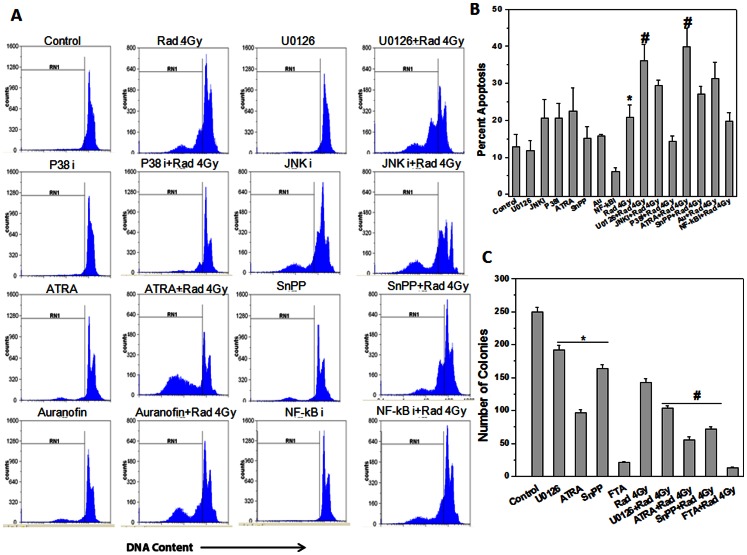
Inhibitors of ERK or Nrf-2 significantly enhanced radiosensitivity of tumor cells. (A) EL-4 cells were incubated with different concentration of pharmacological inhibitors of ERK (10 µM for 2 h) or JNK (10 µM for 2 h) or P38 (10 µM for 2 h) or NF-kB inhibitory peptide (10 µM for 2 h) or Nrf-2 (ATRA) (5 µM for 2 h) or HO-1 (SnPP) (5 µM for 2 h) or TrxRd1 (auranofin) (25 nM for 2 h) or Ras (FTA) (10 µM for 2 h) and were cultured for 48 h in 5% CO_2_ at 37°C with or without exposure to 4 Gy ionizing radiation. Cell death was analyzed by propidium iodide staining and flow cytometry. Representative flow cytometric histograms show apoptotic population as pre-G1 cells. (B) Bar graph represent radiation induced apoptosis in EL-4 cells incubated in the presence of different inhibitors. Data points represent mean±S.E.M. from nine replicates from three independent experiments. *p<0.05, as compared to untreated cells and ^#^p<0.05, as compared to irradiated cells (C) EL-4 cells incubated in the presence of different pharmacological inhibitors were assessed by clonogenic assay for clonogenicity. Bar graph shows number of colonies in different treatment groups. Each bar shows mean±S.E.M from nine replicates from three independent experiments. *p<0.01, as compared to untreated cells and ^#^p<0.01, as compared to irradiated cells.

EL-4 cells were incubated with pharmacological inhibitors of ERK (U0126) or JNK (JNKi) or P38 (P38i) or Nrf-2 (ATRA) or HO-1(SnPP) or thioredoxin reductase (auranofin) or NF-kB inhibitory peptide prior to exposure to 4 Gy and cultured for 48 h. Inhibitors of ERK, Nrf-2, HO-1 and thioredoxin reductase significantly enhanced radiation induced cell death in EL-4 cells suggesting their potential role in cellular radioresistance (Fig. 3A&B). Proliferative potential of EL-4 cells was quantified by clonogenic assay in the presence or absence of these inhibitors. In agreement with previous results, treatment with ERK, Nrf-2, HO-1, thioredoxin reductase or ras inhibitor significantly reduced ability of EL-4 cells to form colonies (Fig. 3C) suggesting their role in cell survival and proliferation.

### EL-4 Cells have Constitutively Active Antioxidant Defense Machinery under Regulation of Nrf-2 that is further Induced by Radiation Exposure

**Figure 4:**
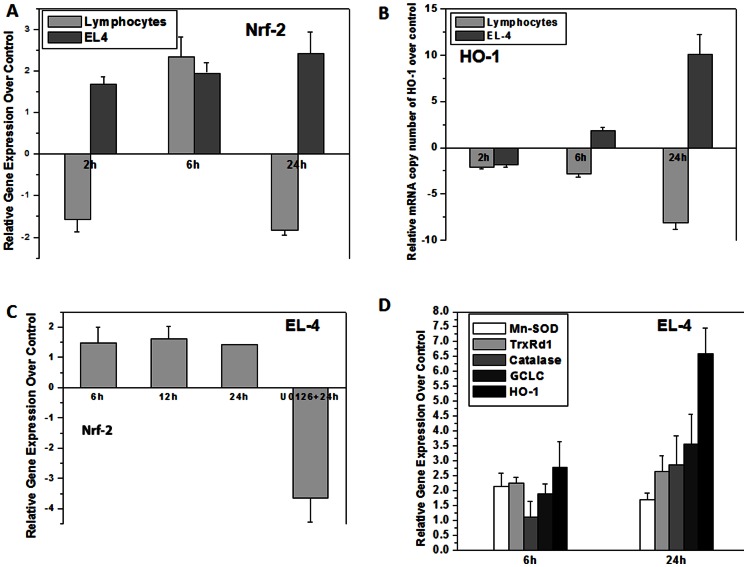
Ionizing radiation induced expression of Nrf-2 and its dependent genes. (A) Quantitative real time PCR for Nrf-2 was performed using total RNA isolated from murine splenic lymphocytes and EL-4 cells exposed to ionizing radiation. Bar graph represents fold change in gene expression of Nrf-2 over control. (B) Data points show relative HO-1 mRNA levels in murine lymphocytes and EL-4 cells post irradiation (4 Gy). (C) mRNA levels of Nrf-2 in EL-4 cells incubated with MEK inhibitor were analyzed. Bar graph shows fold change in gene expression of Nrf-2. (D) Expression of Nrf-2 dependent genes viz. Mn-SOD, catalase, GCLC, thioredoxin reductase and HO-1 was probed by RT-PCR. Bar graph represents mRNA copy number of these genes over control. A total of nine replicates from three independent experiments were used to calculate relative change in gene expression. PCR efficiency for each primer pair was calculated and was used to calculate the fold change in gene expression with respect to control (treatment group and housekeeping gene).

Nrf-2 is essential for the coordinated induction of genes encoding many stress-responsive or cytoprotective enzymes and related proteins [46], [13], [47], [48]. The status of Nrf-2 and its dependent genes was assessed in murine splenic lymphocytes and EL-4 cells exposed to ionizing radiation 4 Gy and harvested at different time intervals (6, 12 & 24 h). Total RNA was extracted by TRIZOL® method and quantitative real time RT-PCR was performed using primers for Nrf-2, HO-1, Mn-SOD, catalase, GCLC and thioredoxin reductase (Table 1). Murine splenic lymphocytes showed complete down regulation of Nrf-2 and HO-1 at 24 hrs but, EL-4 cells showed upregulation in Nrf-2 and HO-1 mRNA levels (Fig. 4A&B). There was a significant increase in mRNA copy number of Nrf-2 dependent genes viz. Mn-SOD, catalase, thioredoxin reductase, GCLC and HO-1. Further, incubation of EL-4 cells with U0126 prior to irradiation resulted in down regulation of Nrf-2 (Fig. 4C).

### Radioresistance of EL-4 Cells is via Activation of Nrf-2 Pathway but Independent of NF-kB

**Figure 5:**
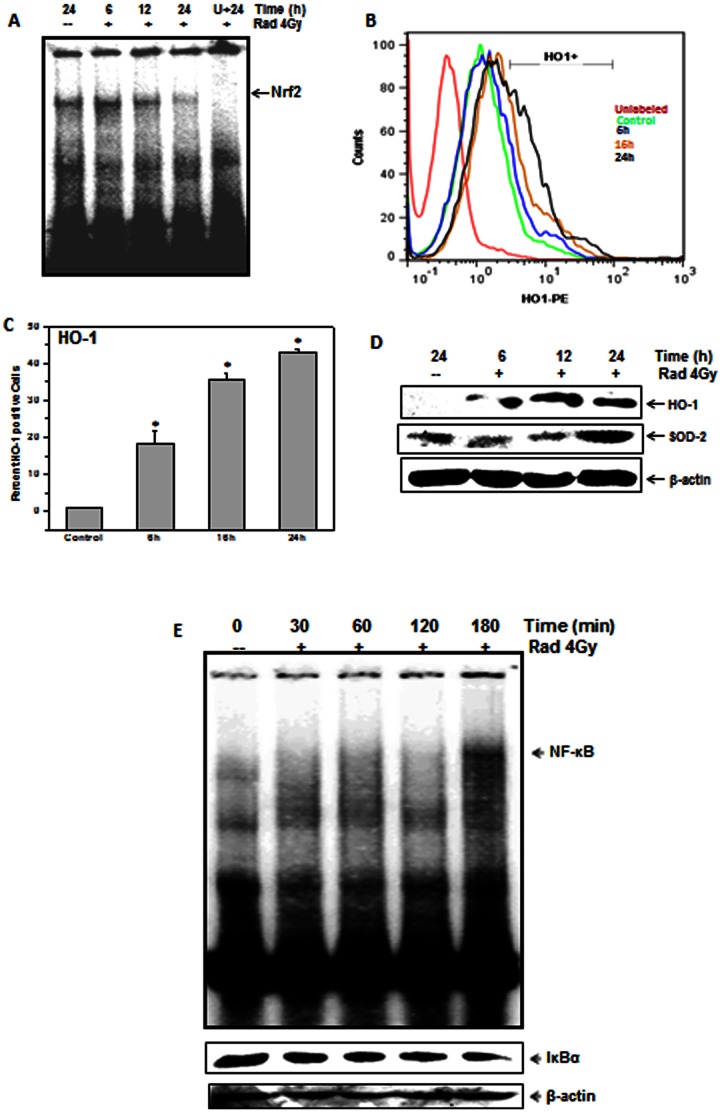
Effect of ionizing radiation on nuclear translocation of Nrf-2 and NF-κB and expression of HO-1 and Mn-SOD. (A) Nuclear levels of Nrf-2 in control and irradiated (4 Gy) EL-4 cells were probed by EMSA in the presence and absence of U0126. (B) Representative flow cytometric histograms show intracellular levels of HO-1. The frequency of PE-HO-1+ cells (gate RN-1) is shown in (C). Bar graph shows percent HO1+ cells. Data points represent mean±S.E.M. from nine replicates from three independent experiments. *p<0.05, as compared to untreated cells. (D) Levels of HO-1 and Mn-SOD in cytosolic extracts were measured by western blotting. (E) Nuclear levels of NF-κB in control or irradiated (4 Gy) EL-4 cells were assessed by EMSA. IκBα degradation at corresponding time points was monitored by western blotting in cytosolic fractions.

Nuclear translocation of Nrf-2 was assessed by EMSA (Fig. 5A). Unirradiated EL-4 cells showed higher levels of Nrf-2 in nuclear extracts suggesting that Nrf-2 is constitutively active. Incubation with U0126 abrogated nuclear translocation of Nrf-2 (Fig. 5A). There is a time dependent increase in HO-1 and Mn-SOD protein levels (Fig. 5B–D). Further, nuclear extracts from EL-4 cells showed no nuclear translocation of NF-kB under normal conditions as seen by EMSA and IkBα degradation (Fig. 5E). Although there was a small increase in nuclear NF-κB at 180 min after radiation exposure, this did not seem to influence the radiosensitivity of EL-4 cells as demonstrated by inhibitory peptide experiment (Fig. 3A and B). Thus, NF-kB did not seem to play a significant role in determining radio resistance of EL-4 cells under these conditions.

### ERK or Nrf-2 Knockdown EL-4 Cells are Significantly more Radiosensitive than Wild Type Cells

**Figure 6:**
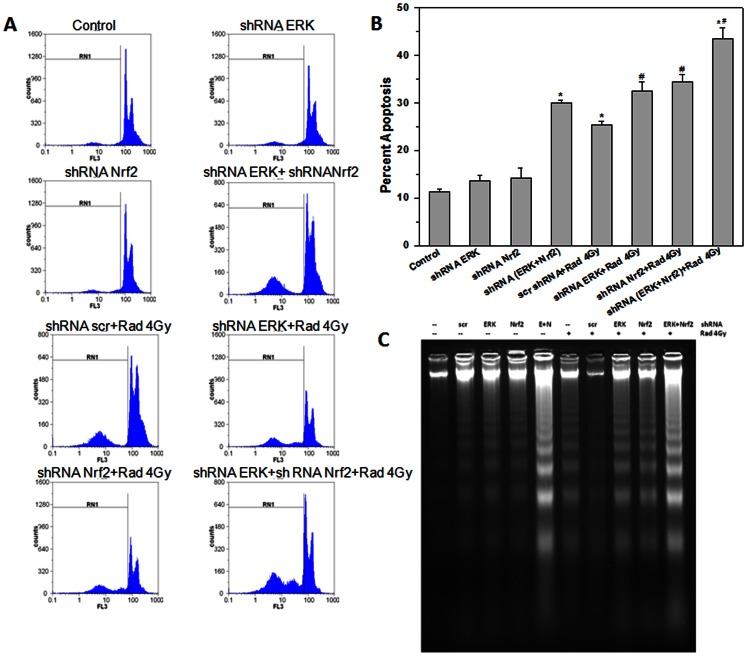
ERK or Nrf-2 knockdown EL-4 cells are significantly more radiosensitive than wild type cells. (A) EL-4 cells transfected with scrambled shRNA or ERK or Nrf-2 shRNA plasmid were exposed to ionizing radiation (4 Gy) and cultured for 48 h. Apoptosis was estimated by propidium iodide staining and flow cytometry. (B) The frequency of apoptotic cells (gate RN1) is shown in the bar graph. Data points represent mean±S.E.M. from nine replicates from three independent experiments. *p<0.05, as compared to unirradiated cells and ^#^p<0.05, as compared to irradiated cells. (C) Genomic DNA from wild type and knock down EL-4 cells cultured for 48 h post radiation exposure or alone was resolved on agarose gel and stained with ethidium bromide. DNA ladder indicates cells undergoing apoptosis.

EL-4 cells were transfected with ERK or Nrf-2 or ERK+Nrf-2 shRNA plasmids and cultured for 48 h. The cells were washed with medium and exposed to 4 Gy ionizing radiation. Two different shRNA plasmids each were used to knock down Erk (shE1 & shE2) and Nrf-2 (shN1 & shN2) respectively. Of these, shE1 for Erk and shN2 for Nrf-2 showed maximum reduction in expression of the target genes and were used in subsequent experiments (Fig. S1). Cells transfected with scrambled shRNA plasmids or vector alone and exposed to radiation served as control. Cell death was measured by PI staining followed by flow cytometry or DNA ladder assay. ERK or Nrf-2 single knockdown cells showed higher radiation induced apoptosis as compared to wild type cells. There was a significant increase in radiation induced apoptosis in ERK and Nrf-2 double knockdown cells as compared to wild type cells or single knock down cells (Fig. 6A&B). DNA fragmentation which is a hallmark of apoptosis was assessed in wild type and knockdown cells post-radiation exposure. These results also confirm that ERK and Nrf-2 are essential for tumor cell survival under normal conditions (Fig. 6C).

## Discussion

Redox homeostasis in tumor and normal cells is constantly disturbed by endogenous and exogenous factors that induce oxidative stress. Normally, ROS levels are maintained below toxic threshold by an inducible antioxidant program that responds to cellular stressors [3]. In normal cells, acute activation of these pathways protect against the formation of mutagenic lesions [7], [49]. But its aberrant activation in premalignant cells may support their survival during inflammation. Somatic mutations that disrupt the Nrf-2-Keap1 interaction to stabilize Nrf-2 and increase the constitutive transcription of Nrf-2 target genes were recently identified in tumor cells [50] [34], indicating enhanced ROS detoxification. In our earlier report we have shown that decreased generation of radiation induced ROS in mouse lymphoma cells was associated with reduced extent of radiation induced apoptosis compared to that in normal lymphocytes [4]. In the present studies we confirmed these observations (Fig. 1) and further investigated role of cellular redox signaling in murine lymphocytes and EL-4 lymphoma cells after exposure to ionizing radiation. This may serve as an informative model to study molecular mechanism of inherent and acquired radioresistance.

Cellular redox balance is sensitive to disruption of intracellular thiol systems. These are sensitive to two electron oxidants and are controlled by the thioredoxin (Trx) and glutathione (GSH). When we measured GSH/GSSG ratio and activity of thioredoxin in EL-4 cells, the ratio of GSH to GSSG was significantly higher in EL-4 lymphoma cells as compared to normal lymphocytes under basal conditions. However, there was a significant decrease in GSH/GSSG ratio in both lymphocytes and EL-4 cells after exposure to ionizing radiation suggesting their involvement in restoration of cellular reduction potential (Fig. 2A). On the contrary, activity of thioredoxin increased significantly in EL-4 cells harvested up to 12 h after exposure to radiation. Increased thioredoxin activity may restore cellular redox equilibrium in EL-4 cells and thus contribute to radioresistance (Fig. 2B).

Cellular redox state can influence the pro-survival/pro-apoptotic signaling targets and thereby decide the fate of a cell [18] [2]. Oxidative stress activates pro-survival signaling molecules such as MAPKs, NF-kB, and Nrf-2 etc. [23]. Hence we used pharmacological inhibitors of these signaling molecules along with radiation in EL-4 cells. Pharmacological inhibitors of MAPK, Nrf-2, HO-1 and thioredoxin reductase significantly enhanced radiosensitivity of EL-4 cells. Interestingly it was observed that, incubation of EL-4 cells with ERK or Nrf-2 inhibitor decreased their cell survival (Fig. 3) and proliferation highlighting their importance in survival of these lymphoma cells. To further confirm these findings, we examined the radiosensitivity of EL-4 cells after knocking down ERK and Nrf-2 individually or in combination. It was observed that knockdown of ERK or Nrf-2 enhanced radiation induced apoptosis (Fig. 6). Further, the double knock down cells were more sensitive to ionizing radiation induced cell death as compared to single knockdown or wild type cells.

Nrf-2 constitutes a unique “redox switch” that can be turned on in response to redox imbalance caused by oxidative and electrophilic stresses [7]–[9]. However, such adaptive response to external stress is normally transient and prone to be readily saturated [8]. The dysregulation of Nrf-2 in cancer has been suggested to protect and offer growth advantages to various cancers and may offer resistance to chemotherapy [27]. EL-4 cells upon radiation exposure showed significant upregulation of Nrf-2 and its dependent genes and protein levels as assessed by quantitative real time RT-PCR, EMSA and Western blot. Nrf-2 was found to be constitutively active in EL-4 cells which may help to express genes involved in scavenging cellular ROS (Fig. 4).

**Figure 7:**
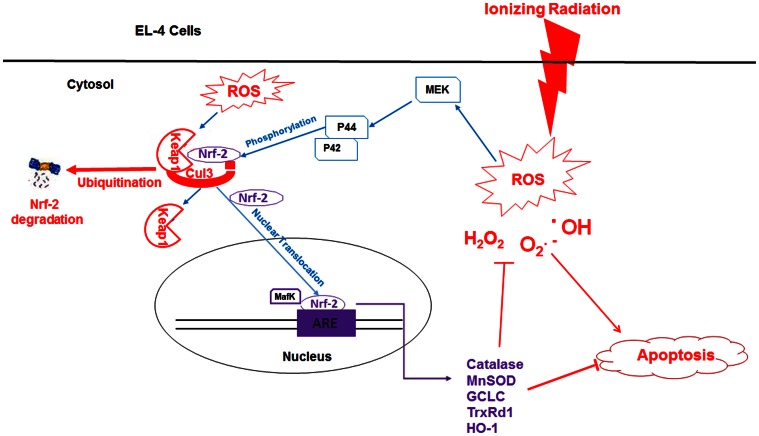
Schematic representation showing the involvement of ERK-Nrf-2 signaling pathway in radioresistance of EL-4 cells.

NF-κB a critical redox sensitive transcription factor is the most intensely investigated molecule in the field of tumor biology, inflammation and radiation oncology [1] [22] [40]. Voluminous literature has underscored the enigmatic role of this molecule in tumor radioresistance which make NF-κB an important target for drug design [1] [21] [23]. This redox sensitive transcription factor has been reported to be constitutively expressed in many tumors [1] [23]. Interestingly, use of NF-kB inhibitory peptide did not enhance radiation induced cell death in EL-4 cells (Fig. 3). On the other hand, recent evidence suggests that activation of Nrf-2 contributes to radioresistance [36]. There are also reports establishing relation between Nrf-2 activation and ionizing radiation and malignant transformation [5] [26] [14] [35] [27] [50] [29]. To our knowledge no direct evidence exists establishing the role of Nrf-2 in differential radiosensitivities of normal and tumor cells. Our results show that constitutive and inducible activation of Nrf-2 and its upstream kinase ERK plays an important role in determining tumor radio-responsiveness. The proposed role of Nrf-2 in radioresistance is depicted in Figure 7. Although, this field is relatively new and unexplored, our findings may provide alternate rationale for drug development to enhance efficacy of tumor radio-therapy.

## Supporting Information

Figure S1 **Reduction in protein expression of ERK or Nrf-2 in EL-4 cells after knockdown using shRNA plasmids.** EL-4 cells were transfected with two different shRNA plasmids each for ERK (shE1 & shE2) and Nrf-2 (shN1 & shN2) as well as scrambled shRNA plasmids. Cells that are not transfected with shRNA plasmid but received all other treatment identical served as mock control. Transfected cells were cultured for 48 h for transgene expression. Cells were treated with 10 µM tert-butylhydroquinone for 10 min for induction of Nrf-2. Whole cell lysates were prepared and probed for Nrf-2 levels by Western blotting. Among the shRNA plasmids used, shE1 for ERK and shN2 for Nrf-2 showed maximum reduction in protein expression. Hence, these two plasmids were used further for all the knockdown experiments.
(TIF)
