# Supplementary figures and images for: Involvement of ERK-Nrf-2 Signaling in Ionizing Radiation Induced Cell Death in Normal and Tumor Cells
Source: PLoS One. 2013 Jun 11;8(6):e65929. doi: 10.1371/journal.pone.0065929 (PMC3679038; doi:10.1371/journal.pone.0065929)

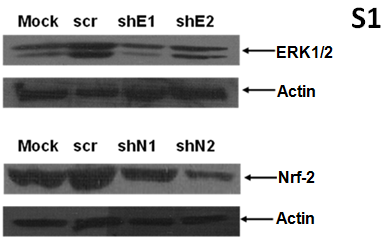

Supplement: Figure S1 — Reduction in protein expression of ERK or Nrf-2 in EL-4 cells after knockdown using shRNA plasmids. EL-4 cells were transfected with two different shRNA plasmids each for ERK (shE1 & shE2) and Nrf-2 (shN1 & shN2) as well as scrambled shRNA plasmids. Cells that are not transfected with shRNA plasmid but received all other treatment identical served as mock control. Transfected cells were cultured for 48 h for transgene expression. Cells were treated with 10 µM tert-butylhydroquinone for 10 min for induction of Nrf-2. Whole cell lysates were prepared and probed for Nrf-2 levels by Western blotting. Among the shRNA plasmids used, shE1 for ERK and shN2 for Nrf-2 showed maximum reduction in protein expression. Hence, these two plasmids were used further for all the knockdown experiments. (TIF) [file pone.0065929.s001.tif]
